# Natural infection of *Lutzomyia longipalpis* (Lutz & Neiva, 1912) by *Leishmania infantum* in a municipality with a high incidence of visceral leishmaniasis in the Brazilian Midwest

**DOI:** 10.1590/0037-8682-0259-2023

**Published:** 2023-09-22

**Authors:** Herintha Coeto Neitzke-Abreu, Georgia Medeiros de Castro Andrade, Paulo Silva de Almeida, Gilmar Cipriano Ribeiro, Thaís Alves Ribeiro, D'Angela Maciel Barrios, Kamily Fagundes Pussi, José Dilermando Andrade, Felipe Dutra-Rêgo, Fredy Galvis Ovallos

**Affiliations:** 1 Universidade Federal da Grande Dourados, Programa de Pós-graduação em Ciências da Saúde, Dourados, MS, Brasil.; 2 Secretaria Municipal de Saúde, Três Lagoas, MS, Brasil.; 3 Universidad Europea Del Atlântico, Santander, Espanha.; 4 Laboratório Regional de Entomologia de Dourados, Secretaria Estadual de Saúde, Dourados, MS, Brasil.; 5 Secretaria Estadual de Saúde, Campo Grande, MS, Brasil.; 6 Instituto René Rachou, Fundação Oswaldo Cruz, Grupo de Estudo em Leishmanioses, Belo Horizonte, MG, Brasil.; 7 Universidade de São Paulo, São Paulo, SP, Brasil.

**Keywords:** Lutzomyia longipalpis, Natural infection, Leishmania infantum, Brazil

## Abstract

**Background:**

Here, *Leishmania* presence in sand flies from Três Lagoas, Mato Grosso do Sul, Brazil, after visceral leishmaniasis (VL) was investigated.

**Methods:**

In April 2022, two light traps were deployed within and around the residence for two days post-VL case report.

**Results:**

A total of 120 *Lutzomyia longipalpis* were collected. Suprapyloric flagellates were found in a female sand fly with eggs and residual blood during midgut dissection. Sequencing of ITS1 and *cytb* fragments confirmed *Leishmania infantum* DNA and identified *Homo sapiens* as the blood source, respectively.

**Conclusions:**

This study emphasizes the importance of monitoring sand flies in VL endemic areas.

Visceral leishmaniasis (VL) is a severe disease caused by the protozoan parasite *Leishmania infantum* that affects both humans and other mammals on a global scale^1^. In Brazil, VL is a significant public health challenge that notably affects the northeastern and southeastern regions of the country^2^. The disease is transmitted through the bites of infected female sand flies, with domestic dogs serving as the parasite's primary reservoir, posing complex obstacles to disease control^1^. 

Mato Grosso do Sul state (MS) is an endemic area for VL. Nonetheless, until the early 1990s, the disease was confined to the geographical region of Corumbá and Ladário municipalities located along the border with Bolivia^3^. Since 2000, the municipality of Três Lagoas has witnessed recurrent urban VL outbreaks, registering nearly 30 autochthonous cases within the preceding three years, leading to elevated fatality rates^3^ ^,^ ^4^. The recent upsurge in VL outbreaks and heightened mortality rates within the municipality have spurred apprehensions among Brazilian authorities, with a cumulative tally of 27 human cases documented in the preceding three-year period^2^ ^,^ ^4^. Moreover, it is noteworthy that the distribution of VL occurrences spans both urban neighborhoods and rural zones of Três Lagoas, where the year-round presence of sand fly vectors has been well-documented^4^.

The role of *Lutzomyia longipalpis* as the main vector of *Le. infantum* is widely acknowledged in Brazil, with this species exhibiting a broad distribution across geographic regions, coinciding with instances of autochthonous VL^5^. Furthermore, the capacity of *Lu. longipalpis* to sustain late-stage infections involving diverse *Leishmania* species has been recognized, and prior documentation has affirmed the natural infection of this species with *Le. infantum* ^6^. However, the prevailing strategies for VL control have proven ineffective, culminating in Três Lagoas becoming the second-most afflicted municipality regarding reported cases^2^. Consequently, there is a recommendation for heightened entomological surveillance activities within locales reporting human cases to detect local transmission and instigate pertinent control measures. Thus, the focal aim of this study was to delineate the outcomes of entomological surveillance triggered by a report of human VL within the confines of the Três Lagoas Municipality.

A suspected case of autochthonous human VL was reported during local communicable disease surveillance in April 2022. The afflicted individual was a resident of the Jardim Novo Aeroporto neighborhood. Despite Três Lagoas municipality (20°45'35'' S, 51°41'42'' W), situated within the state of MS, Brazil (Figure 1), being an established endemic region for VL, efforts to substantiate the autochthonous nature of this particular case were undertaken. This approach was aligned with the guidelines provided by the National Program for the Prevention and Control of Visceral Leishmaniasis. The sand fly collection was conducted using light traps for two nights in April 2022 in the intradomicile and peridomicile areas, where dogs and a chicken coop were observed. All insects were separated and transported live in rearing boxes to the field laboratory for midgut dissection and taxonomic identification.

**FIGURE 1:**
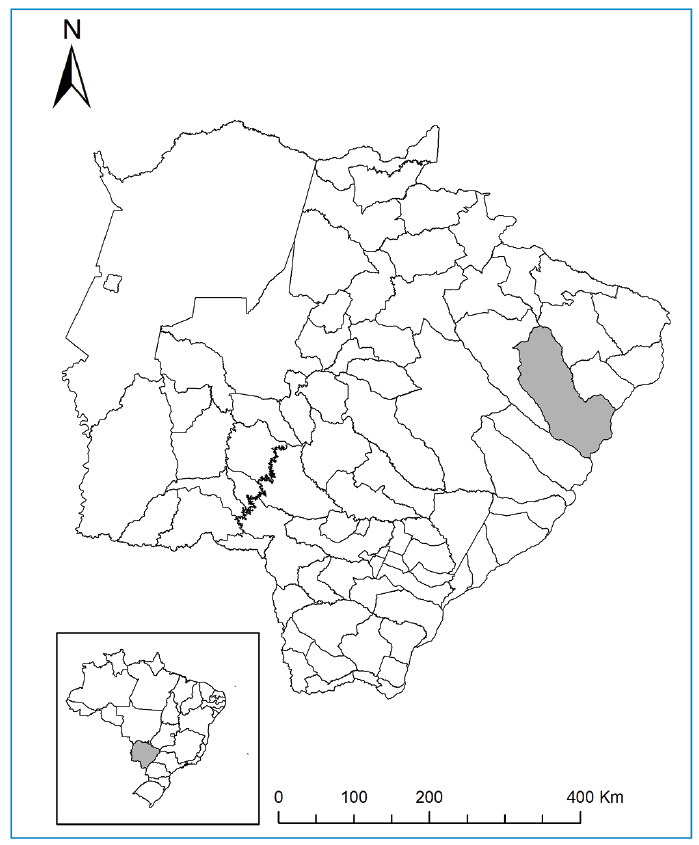
Location of the collection site within Três Lagoas, Mato Grosso do Sul, Brazil.

Sand flies were promptly processed at the Entomology Laboratory of the Zoonosis Control Center of the municipality. Female specimens were dissected using sterile needles to expose the midgut, and the presence of flagellate forms was evaluated using optical microscopy (at 400x and 1000x magnification). Furthermore, the sand flies were identified based on morphological characteristics^7^. For positive samples, the midguts were triturated and preserved in 1.5mL tubes containing 1x phosphate-buffered saline (pH 7.2) at -20°C until DNA extraction. Whole DNA was extracted using 5% Chelex solution. Moreover, the presence of *Leishmania* was monitored through PCR using the 13A/13B primers^8^ and ITS1-PCR^9^, while species identification was achieved via Sanger sequencing. PCR was performed using the reference strains of *Le. infantum* (MHOM/BR/1974/PP75) and *Le. braziliensis* (MHOM/BR/1975/M2903) were used as the positive and negative controls, respectively.

DNA extracted from the female displaying blood in the midgut underwent PCR targeting the *cytb* gene to identify the source of the blood^10^. Within the PCR, DNA extracted from *Gallus gallus* served as the positive control, whereas DNA from male sand flies was used as the negative control. Following a positive PCR result, the product was purified, subjected to Sanger sequencing, and subsequently analyzed by comparison with sequences cataloged in GenBank database^11^.

In the state of MS, *Lu. Longipalpis* has been documented in at least 44 municipalities, including Três Lagoas, where it has emerged as the predominant species within both intra and peridomiciliary environments^12^ ^,^ ^13^. In this study, 120 sand flies were collected, all belonging to *Lu. longipalpis*. Specifically, three specimens were collected in the intradomicile (two males and one female), whereas the remaining 117 specimens were collected in the peridomicile (84 males and 33 females). All female specimens were dissected immediately to expose their midguts. Notably, during the dissection of a female from the peridomiciliary area, seemingly toward the conclusion of the gonotrophic cycle (as evidenced by the presence of mature eggs and remnants indicative of blood digestion), suprapyloric promastigote forms were detected (Supplementary File 1). Furthermore, 13A/13B (Figure 2) and ITS1 PCRs confirmed the presence of *Leishmania*, and DNA sequencing of the ITS1 amplicon (300-350bp) allows the identification of these flagellates as *Le. infantum* (GenBank accession number OQ675107). 

**FIGURE 2:**
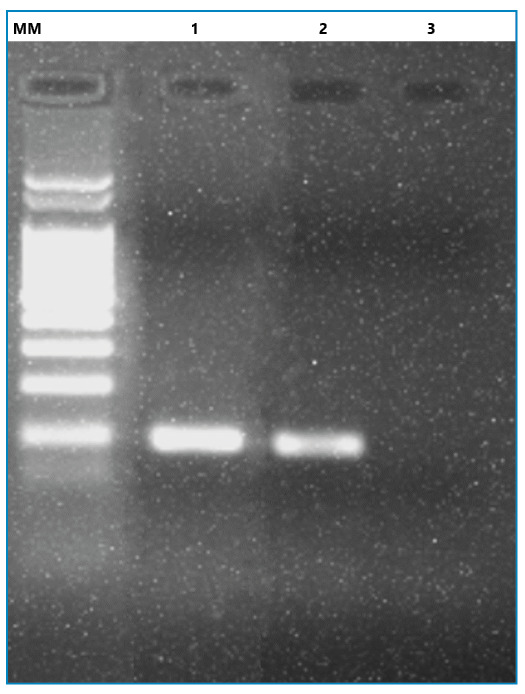
Representative agarose gel of molecular identification of *Leishmania* in *Lutzomyia longipalpis* from Três Lagoas, Mato Grosso do Sul, Brazil. Lanes: MM, molecular weight marker (100 bp); 1, sand fly sample; 2, *Leishmania braziliensis* (MHOM/BR/1975/M2903) strain used as positive control; 3, non-template control.

Considering that the female sand fly displaying *Leishmania* infection demonstrated residual blood remnants indicative of recent feeding, a *cytb*-PCR analysis was performed to ascertain the source of this blood meal. DNA sequencing indicated the presence of human (*Homo sapiens*) DNA in the blood sample, with an identity match exceeding 99%. Notably, no other female specimens displayed discernible indications of recent blood ingestion.

The described results support the determination of autochthony in VL cases and the implementation of the following control activities (i.e., residual insecticide application in residences and dog surveys). Notably, ecological parameters related to vector-host interactions are key to understanding vector capacity^14^. The condition of the female at the end of the gonotrophic cycle suggested that it was close to oviposition and was searching for a new blood meal. Furthermore, the presence of human blood in the intestine suggests that this female may have attempted to feed on humans of significant epidemiological importance, considering the state of infection with flagellates. 

The circulation of an infected female highlights the need for constant monitoring of this zoonosis in the study area, particularly to intensify measures to control canine reservoirs and vectors. Effective prevention and control of VL require a thorough understanding of the epidemiology and ecology of the disease, including the role of vector insects in its transmission. Thus, the results presented in this report reinforce the importance of entomological surveillance activities as support for prevention and control programs for zoonosis, providing evidence for identifying risk areas to support public health decision-making. 
